# Mental health assessment of the Brazilian LGBTQIAP+ population during the COVID-19 pandemic: a cross-sectional study

**DOI:** 10.1590/1980-220X-REEUSP-2023-0148en

**Published:** 2023-09-04

**Authors:** Thayane Martins Dornelles, Emerson Silveira de Brito, Ben Hur Graboski Pinheiro, Bianca de Morais Cunha Santarem, Sofia Silva Torres Aguer, Analuiza Camozzato

**Affiliations:** 1 Universidade Federal de Ciências da Saúde de Porto Alegre, Porto Alegre, RS, Brazil.; 2 Universidade Federal do Rio Grande do Sul, Porto Alegre, RS, Brazil.

**Keywords:** COVID-19, Sexual and Gender Minorities, Mental Health, Healthcare Disparities, COVID-19, Minorias Sexuais e de Gênero, Saúde Mental, Disparidades em Assistência à Saúde, COVID-19, Minorías Sexuales y de Género, Salud Mental, Disparidades en Atención de Salud

## Abstract

**Objective:**

To investigate the association between the frequency and associated factors of non-psychotic mental disorders and anxiety symptoms in a Brazilian LGBTQIAP+ sample.

**Method:**

Cross-sectional study, conducted from September to October 2020 using an online questionnaire, with instruments for sociodemographic characterization, the Generalized Anxiety Disorder Screener and the Self-Report Questionnaire. The analysis was performed using the Chi-square and Fisher’s Exact tests. Poisson regression with robust variance was performed to estimate the effect of sociodemographic variables on mental health.

**Results:**

Positive screening for anxiety and non-psychotic disorders were identified in 85.2% and 60.2% of the participants, respectively. Younger age groups, who professed some religion, only had access to public health, and presented with medical conditions showed a higher risk for non-psychotic mental disorders. Individuals under 30 (1.33, 95%, CI = 1.17–1.52) presented a high risk for anxiety symptoms.

**Conclusion:**

The prevalence of anxiety and non-psychotic disorders during the COVID pandemic was high. Implementation of health policies and interventions targeting identified risk factors is recommended.

## RESUMO

**Objetivo:**

Investigar a associação entre a frequência e fatores associados de transtornos mentais não psicóticos e sintomas de ansiedade em uma amostra brasileira LGBTQIAP+.

**Método:**

Estudo transversal, realizado de setembro a outubro de 2020 por meio de um questionário online, incluindo instrumentos de caracterização sociodemográfica, *Generalized Anxiety Disorder Screener* e o *Self-Report Questionnaire*. A análise foi realizada utilizando-se testes Qui-quadrado e Exato de Fisher. Regressão de Poisson com variância robusta foi realizada para estimar o efeitodas variáveis sociodemográficas sobre a saúde mental.

**Resultados:**

A triagem positiva para ansiedade e transtornos não psicóticos foi identificada em 85,2% e 60,2% dos participantes, respectivamente. Faixas etárias mais jovens, que possuíam alguma religião, tinham acesso somente à saúde pública e alguma condição clínica prévia apresentaram maior risco para transtornos mentais não psicóticos. Indivíduos com menos de 30 anos (1,33, 95%, IC = 1,17–1,52) apresentaram alto risco para sintomas de ansiedade.

**Conclusão:**

A prevalência de ansiedade e transtornos não psicóticos durante a pandemia de COVID foi alta. Recomenda-se a implementação de políticas e intervenções de saúde direcionadas aos fatores de risco identificados.

## RESUMEN

**Objetivo:**

Investigar la asociación entre la frecuencia y los factores asociados de trastornos mentales no psicóticos y síntomas de ansiedad en una muestra brasileña LGBTQIAP+.

**Método:**

Estudio transversal realizado de septiembre a octubre de 2020 mediante cuestionario en línea, con instrumentos de caracterización sociodemográfica, el *Generalized Anxiety Disorder Screener* y el *Self-Report Questionnaire*. El análisis se realizó mediante las pruebas Chi-cuadrado y Exacto de Fisher. Se realizó una regresión de Poisson con varianza robusta para estimar el efecto de las variables sociodemográficas sobre la salud mental.

**Resultados:**

Se observó resultado positivo para ansiedad y trastornos no psicóticos en el 85,2% y el 60,2% de los participantes, respectivamente. Los grupos de edad más jóvenes, que profesaban alguna religión, solo tenían acceso a la salud pública y tenían condiciones médicas presentaban mayor riesgo de trastornos mentales no psicóticos. Los individuos menores de 30 años (1,33, 95%, IC = 1,17–1,52) presentaron alto riesgo de síntomas de ansiedad.

**Conclusión:**

La prevalencia de ansiedad y trastornos no psicóticos durante la pandemia de COVID fue alta. Se recomienda la implementación de políticas e intervenciones de salud dirigidas a los factores de riesgo identificados.

## INTRODUCTION

On March 11^th^, 2020, the World Health Organization declared the novel coronavirus disease called “COVID-19” a pandemic. Until March 10th, 2023, 6.881.955 deaths were recorded worldwide, and out of these, 699.310. 000 were in Brazil alone^(1)^. Governments have promoted changes in lifestyle habits to contain the pandemic, including social isolation, remote learning, work from home, and changes on the way people interact with each other^(2)^.

Prior to the COVID-19 pandemic, an increase in the prevalence of mental illnesses was detected, and the WHO (2017) estimated that 322 million adults worldwide would experience some type of mental disorder. In Brazil, an epidemiological study conducted by the Ministry of Health indicated a prevalence of mental disorders of around 20% in the adult population before the pandemic^(3)^. Furthermore, depression, anxiety, substance use, and an increase in suicides can occur following natural disasters, pandemics, and economic crises^(4,5)^.

Many studies have identified a high prevalence of depression and anxiety associated with COVID-19 and its consequences in the general population, including the LGBTQIAP+ community. A meta-analysis of 221,970 participants evidenced the prevalence of depression (31.4%), anxiety (31.9%), and distress (41.1%) during the COVID-19 pandemic^(2)^. In comparison, the prevalence of mental distress, anxiety, and depression in this same period was almost twice as high in the LGBTQIAP+ population^(6)^.

In this context, research studies have consistently elucidated mental health disparities among LGBTQIAP+ individuals when compared with their heterosexual, cisgender counterparts^(7)^. These disparities were related to social inequalities that disproportionately affect the LGBTQIAP+ community. For instance, a greater proportion of these individuals lack access to health insurance (17% vs. 12%) and face poverty (22% vs. 16%) when compared to their non-LGBTQIAP+ counterparts. Same-sex parents and single LGBTQIAP+ parents are at least twice as likely to live near the poverty line compared to their non-LGBTQIAP+ peers^(8)^. Therefore, COVID-19 can exacerbate social inequalities^(9)^.

The minority stress model has been the main explanation for health disparities among sexual minorities. This model is a conceptual theory that describes the stress processes related to the stigma and prejudice experienced by individuals belonging to this group. The stressors include enacted stigma (persecution, rejection, aggression, violence), internalized homonegativity (shame, denial and self-destructive behavior), and concealment of sexual identity (attempts to hide one’s own sexuality)^(10)^.

Considering the invisibility, stigma, discrimination, and social inequalities to which LGBTQIAP+ population is exposed, which were intensified during the pandemic, it is necessary to identify the potential damage caused to this population’s mental health - this will provide information for planning specific health policies and interventions. The present study aimed to investigate the association between the frequency and associated factors of non-psychotic mental disorders and anxiety symptoms in a Brazilian LGBTQIAP+ sample.

## METHOD

### Study Design

This is a descriptive, analytical, and cross-sectional study, developed according to the guidelines of the Strengthening the Reporting of Observational Studies in Epidemiology (STROBE) and the CHERRIES (The Checklist for Reporting Results of Internet E-Surveys), which guided the report of this study^(11,12)^.

### Population

The population consisted of individuals from the gay, lesbian, bisexual, and other gender minorities aged over 18 years old who were invited to answer an online questionnaire.

### Local

This study was conducted in several Brazilian states, online, during the period of social isolation.

### Selection Criteria

The inclusion of the participants was through invitations on social media (Instagram, WhatsApp, and Facebook) and also by contacting representatives of the LGBTQIAP+ population in Brazil. To participate in the research, it was necessary to be over 18 years old and self-identify as LGBTQIAP+. The snowball technique was used in this study, where participants who received the invitation were also asked to share it with other individuals from their same category, using social media^(13)^. This method resulted in a non-probabilistic convenience sample. The participants who answered less than 20% of the questionnaire were excluded.

### Sample Size

The sample size was calculated at 315 individuals considering a 95% confidence level, based on an 8% prevalence of depression in the community and a minimum difference of 3% in prevalence. Considering a 20% loss rate, the necessary sample size was corrected to 378 individuals^(14)^. A pilot study was conducted with the first 20 participants to assess communication gaps and the continuity of the questions. The participants of the pilot study were included in the sample. Some questions were adjusted after observations and suggestions from these respondents to improve data quality.

### Data Collection

The semi-structured questionnaire was developed using REDcap electronic data capture tools^(15)^. The instrument included sociodemographic information: age, gender assignment, sexual orientation (gay, lesbian, bisexual, or other), gender identity (cisgender, transgender, non-binary or other), race/skin color (white, black/brown), relationship status (single, dating, or married/living with a partner), religion, education level (secondary education level: finished or unfinished, and higher education level: finished or unfinished undergraduate course), employment status (formal: work with signed contract, informal: self-employed work, unemployed or student), access to health service (public or private). Possible medical conditions that the participant could present were also included in the questionnaire according to the Centers for Disease Control and Prevention (CDC)^(16)^ classification (chronic disease, moderate to severe asthma, cardiovascular diseases, cancer, bone marrow transplant, use of immunosuppressive medications, kidney disease on dialysis, liver failure, severe hypertension, and diabetes), HIV status, and the Generalized Anxiety Disorder Screener (GAD-7) and Self-Report Questionnaire (SRQ-20) mental health self-reported screening scales^(17,18)^.

GAD-7 is a brief instrument for the assessment, diagnosis, and monitoring of anxiety. It consists of seven items, arranged on a four-point scale: from 0 (not at all) to 3 (almost every day), with scores ranging from 0–21, measuring the frequency of anxiety symptoms and signs over the past two weeks. The cutoff points were 5, therefore the results were classified as none/normal (0–4 points), mild (5–9), moderate (10–14), and severe anxiety (15–21 points). The instrument has an excellent Cronbach alpha internal consistency score (0.880)^(18)^.

SRQ-20 is a screening instrument. It is aimed at detecting symptoms, suggesting a level of suspicion (presence/absence) of a mental disorder, but does not indicate a specific diagnosis. The evaluated symptoms are similar to common mental disorders, which are characterized by non-psychotic symptoms, such as insomnia, fatigue, irritability, forgetfulness, difficulty concentrating, and somatic complaints. The questionnaire consists of 20 dichotomous questions (yes and no), four about physical symptoms and 16 about psychological and emotional disorders. The scores obtained vary from 0 (no probability) to 20 (extreme probability). Total scores equal to or greater than seven are considered positive screening. SRQ-20 internal consistency was assessed using Cronbach’s alpha (α) and presented internal consistency index (α = 0.86), with 86.33% sensitivity and 89.31% specificity^(17)^.

### Data Analysis and Treatment

The categorical variables were summarized using absolute frequencies and the continuous variables, through mean and standard deviation. The Chi-square and Fisher’s Exact tests were used to compare proportions and the t-test or equivalent non-parametric test was used for the continuous variables. For all analyses, a 5% significance level and 80% power were adopted. The variables that showed association in the bivariate model were included in the multivariate model. A multivariate model was performed using Poisson regression with robust variance, adjusted to age, sexual orientation, religion, access to health services, and medical condition. The statistical analysis was carried out in the Statistical Package for the Social Sciences (SPSS) software, version 26.0.

### Ethical Aspects

The project was approved by the Research Ethics Committee of the Universidade Federal de Ciências da Saúde de Porto Alegre, (UFCSPA), number 4.270.572. All ethical procedures were adopted considering the current Brazilian legislation in the Regulatory Guidelines and Standards for Research involving Human Beings (Resolution 466/12) and in accordance with the Declaration of Helsinki^(19)^. All participants consented to participate in the study by selecting the checkbox and had the option to download the form sheet.

## RESULTS

A final sample of 655 participants answered the questionnaire. According to sexual orientation, they self-declared as gay (58.9%), lesbian (15.6%), bisexual (21.7%), or other sexual and gender minorities (3.8%). The mean age was 29.7 (+8.7) years old. Most of the participants presented tertiary or higher education level (92.1%), white skin color (74.6%), no religion (56%), access to private health services (74.8%), and formal work (55.3%). Half of the participants were single (51.4%) and the majority was cisgender (93.9%). The presence of medical conditions and HIV infection was reported in 20.4% and 9.5% of the sample, respectively. Age, schooling level, relationship status, labor market, and presence of at least one medical condition were significantly different according to sexual orientation (p < 0.01) (Table 1).

**Table 1:** Sociodemographic characteristics according to sexual orientation in the Brazilian LGBTQIAP+ population – Porto Alegre, RS, Brazil, 2020.

Characteristics	Total	Sexual orientation				p-value^a^
		Gay (n = 386) n (%)	Lesbian (n = 102) n (%)	Bisexual (n = 142) n (%)	Other^*^ (n = 25) n (%)	
**Mean age (SD)**	29.7 (8.7)	31.6 (8.4)	29.7 (9.5)	24.4 (5.5)	32.4 (12)	<0.01
**Age**						<0.01
18–30 years old	414 (62.3)	201 (52.1)	68 (66.7)	130 (90.3)	15 (57.7)	
31–40 years old	170 (25.8)	135 (35)	20 (19.6)	10 (6.9)	5 (19.2)	
> 40 years old	74 (11.2)	50 (13)	14 (13.7)	4 (2.8)	6 (23.1)	
**Schooling level**						<0.01
High School or lower	52 (7.9)	19 (4.9)	6 (5.9)	17 (11.8)	10 (38.5)	
Tertiary or higher	606 (92.1)	367 (95.1)	96 (94.1)	127 (88.2)	16 (61.5)	
**Gender identity**						**—**
Cisgender	615 (93.9)	96 (95.6)	132 (94.1)	19 (93)	19 (73.1)	
Transgender	11 (1.7)	3 (0.8)	0	4 (2.8)	4 (15.4)	
Non-binary	29 (4.4)	14 (3.6)	6 (5.9)	6 (4.2)	3 (11.5)	
**Race/Ethnicity**						0.8
White	491 (74.6)	285 (75)	78 (77.2)	114 (79.7)	14 (56)	
Black/Brown	158 (24.3)	95 (25)	23 (22.8)	29 (20.3)	11 (44)	
**Religion**						0.06
Yes	289 (44)	186 (48.2)	42 (41.8)	51 (35.4)	10 (40)	
No	368 (56)	200 (51.8)	60 (58.8)	93 (64.6)	25 (60)	
**Relationship status**						<0.01
Single	338 (51.4)	223 (57.8)	34 (33.3)	72 (50)	9 (34.6)	
Dating	161 (24.5)	77 (19.9)	32 (31.4)	49 (34)	3 (11.5)	
Married	159 (24.1)	86 (22.3)	36 (35.3)	23 (16)	14 (52.8)	
**Access to health services**						0.8
Public	165 (25.2)	140 (38.1)	37 (37.8)	56 (40.9)	11 (45.8)	
Private	490 (74.8)	227 (61.9)	61 (62.2)	81 (59.9)	13 (54.2)	
**Employment status**						<0.01
Formal	363(55.3)	244 (63.4)	58 (56.9)	51 (35.4)	10 (38.5)	
Informal	108 (16.4)	66 (17.1)	13 (12.7)	21 (14.6)	8 (30.8)	
Unemployed	48 (7.3)	31 (8.1)	3 (2.9)	10 (6.9)	4 (15.4)	
Student	138 (21)	44 (11.4)	28 (27.5)	62 (43.1)	4 (15.4)	
**Medical condition**	161 (20.4)	97 (60.2)	33 (20.5)	27 (16.8)	4 (2.5)	0.72
**HIV** ^b^ **-positive**	57 (9.5)	56 (15.3)	1 (1)	0	0	**—**

^*^Other: asexual, demisexual and pansexual.

^a^Chi-square test.

Analysis of variance (ANOVA). SD, standard deviations;

^b^HIV, Human immunodeficiency virus.

From the total of 655 participants, 394 (60.2%) screened positive for non-psychotic disorders according to SRQ-20.

The bivariate analyses showed higher anxiety levels in those participants from lower age groups, with bisexual orientation, without religion, and who had public health assistance. Non-psychotic mental disorders screened by the SRQ-20 scale were associated with lower age, bisexual orientation, absence of religion, access to public health services, presence of at least one medical condition, and positive HIV (Table 2).

**Table 2:** Generalized Anxiety Disorder Scale and Self-Report Questionnaire Scale results according to sociodemographic characteristics in the LGBTQIAP+ population – Porto Alegre, RS, Brazil, 2020.

	GAD-7		p-value^a^	SRQ-20		p-value^a^
	Negative (0–4) n (%)	Positive (5–21) n (%)		Negative n (%)	Positive n (%)	
**Age**			<0.01			<0.01
< 30 years old	43 (11.1)	345 (88.9)		125 (32)	266 (68)	
31–40 years old	24 (15.7)	129 (84.3)		71 (43.5)	85 (54.5)	
> 40 years old	22 (37.3)	13 (22.0)		44 (72.1)	17 (27.9)	
**Schooling level**			0.13			0.2
High School or lower	3 (7)	40 (93.0)		13 (29.5)	31 (70.5)	
Tertiary or higher	86 (15.4)	471 (84.6)		227 (40.2)	337 (59.8)	
**Sexual orientation**			0.17			<0.01
Gay	59 (16.8)	292 (83.2)		164 (46.1)	192 (53.9)	
Lesbian	14 (14.6)	82 (85.4)		35 (35.7)	63 (64.3)	
Bisexual	13 (10)	117 (90.0)		34 (26.0)	97 (74.0)	
**Race/Ethnicity**			0.58			0.28
White	65 (14.4)	387 (85.6)		176 (38.5)	281 (61.5)	
Black/Brown	24 (16.2)	124 (22.3)		62 (43.7)	80 (56.3)	
**Religion**			0.19			<0.01
Yes	44 (17.0)	215 (83.0)		127 (48.8)	135 (51.5)	
No	45 (13.2)	296 (86.8)		113 (32.7)	233 (67.3)	
**Relationship status**			0.12			0.60
Single	45 (14.8)	260 (85.2)		126 (40.6)	184 (59.4)	
Dating	16 (10.7)	133 (89.3)		54 (36.0)	96 (64.0)	
Married	28 (19.2)	118 (80.8)		60 (40.5)	88 (59.5)	
**Access to health services**			0.46			0.04
Public	31 (13.5)	199 (86.5)		80 (34.2)	154 (65.8)	
Private	58 (15.7)	312 (84.3)		160 (42.8)	214 (57.4)	
**Employment status**			0.03			0.25
Formal	55 (16.8)	273 (83.2)		140 (42.2)	192 (57.8)	
Informal	19 (19.4)	79 (80.6)		40 (40.0)	60 (60.0)	
Unemployed	4 (8.9)	41 (91.1)		13 (28.9)	32 (71.1)	
Student	10 (7.8)	118 (92.2)		46 (35.4)	84 (64.6)	
**Medical condition**			0.16			<0.01
No	73 (15.3)	476 (84.7)		208 (43.0)	276 (57)	
Yes	16 (12.9)	108 (87.1)		32 (25.8)	92 (74.2)	
**HIV-positive**			0.49			<0.01
No	78 (14.3)	468 (85.7)		31 (57.4)	23 (42.6)	
Yes	11 (20.4)	43 (79.6)		209 (37.7)	345 (62.3)	

^a^Chi-square test.

GAD-7, Generalized Anxiety Disorder Scale; SRQ-20, Self-Report Questionnaire Scale; HIV, Human immunodeficiency virus.

In the multivariate model, participants under 30 (PR = 1.33, 95% CI = 1.17–1.52) had at a higher risk of presenting anxiety symptoms according to the GAD-7 scale, as well as those with access only to public health services (PR = 1.13, 95% CI = 1.04–1.22) and with at least one medical condition (PR = 1.13, 95% CI = 1.03–1.24). The participants belonging to lower age groups (PR = 1.49, 95% CI = 1.32–1.63), who professed a religion (PR = 1.12, 95% CI = 1.03–1.21), with access to public health services (PR = 1.08, 95% CI = 1.01–1.16), and with at least one medical condition (PR = 1.26, 95% CI = 1.16–1.37) presented a higher risk for non-psychotic mental disorders (Table 3).

**Table 3:** Adjusted prevalence ratio of the association scales Generalized Anxiety Disorder Scale and Self-Report Questionnaire and independent variables of the study in the LGBTQIAP+ population – Porto Alegre, RS, Brazil, 2020.

Variables	Anxiety (GAD-7)			Mental disorder symptoms (SRQ-20)	
	PR	95% CI		PR	95% CI
**Age**					
> 40	1			1	
≤ 30	1.33	1.17–1.52		1.49	1.32–1.63
31–40	1.27	1.10–1.47		1.40	1.32–1.69
**Sexual orientation**					
Lesbian	1			1	
Gay	0.93	0.83–1.04		0.92	0.83–1.01
Bisexual	0.53	0.92–1.18		01.05	0.93–1.17
**Religion**					
No	1			1	
Yes	01.03	0.95–1.12		1.12	1.03–1.21
**Access to health services**					
Private	1			1	
Public	1.13	1.04–1.22		01.08	1.01–1.16
**Medical condition**					
No	1			1	
Yes	1.13	1.03–1.24	1.26		1.16–1.37

Multivariate model adjusted to age, sexual orientation, religion, access to health services and medical condition.

GAD-7, Generalized Anxiety Disorder Scale; SRQ-20, Self-Report Questionnaire Scale; CI, confidence interval; PR, Prevalence Ratio.

## DISCUSSION

This was the first study that evaluated the impacts of the COVID-19 pandemic using the SRQ-20 and GAD-7 scales in the Brazilian LGBTQIAP+ population. We found very high prevalence values for non-psychotic disorders and anxiety during the first quarantine period. Younger age, bisexual sexual orientation, access only to public health care, and presence of at least one medical condition were associated both to anxiety and non-psychotic disorders. The participants who professed no religion presented a high prevalence of non-psychotic disorders.

We found that the prevalence of anxiety and non-psychotic symptoms in our study can be linked to the historical context of this population, which has been aggravated by the pandemic. Preceding the COVID-19 pandemic, 67% of the LGBTQIAP+ youth reported facing family rejection, 77% reported feeling depressed in the past week, and 95% reported sleep-related disturbances^(20)^. The prevalence of mental health problems during the COVID-19 pandemic in the general population was 31.5%^(2)^. In the global LGBTQIAP+ community, 51.4% of the individuals reported moderate to severe psychological distress (18.0% moderate, 31.4% severe), 36.4% were positive for anxiety, and 41.6% were positive for depression^(21)^. A study conducted in Brazil compared the prevalence of mental health problems in LGBTQIAP+ and non-LGBTQIAP+ individuals, finding a rate of 51.95% in LGBTQIAP+ vs. 32.70% in cisgender heterosexuals, while the rates for anxiety disorders were 30.14% vs. 13.37% and, for depressive disorders, they were 27.75% vs. 15.34%^(22)^. These prevalence rates were higher than those observed for the general population and in other LGBTQIAP+ studies^(20–24)^.

A number of surveys indicated that, when compared to heterosexual adults, LGBTQIAP+ individuals are 2 to 5 times more likely to have substance use problems, 2 times more likely to have anxiety and mood disorders, and present 2.5 times higher risk of suicide^(22,23)^. Before the COVID-19 pandemic, the LGBTQIAP+ population had high prevalence of mental disorders when compared to heterosexual people^(24)^. The literature establishes that social determinants directly influence health status and quality of life^(23)^. Discrimination and rejection add up to daily stressors, compromising the mental health of the LGBTQIAP+ population. These can be considered important elements of social determinants in sexual and gender minority populations, that potentiate health problems in these groups^(23,25)^. In addition, low family support, homophobia by family members, violence, rejection, substance abuse, shame, and isolation are associated with mental health problems^(24)^.

Another finding of the study was that younger LGBTQIAP+ individuals showed higher rates of anxiety and other ­non-psychotic disorders. It is already known that younger people and sexual and gender minorities are more likely to develop mental health-related problems and, in the general population, young people had higher rates of psychological distress during the COVID-19 pandemic^(20)^. Older people from the general or LGBTQIAP+ populations may have greater resilience when faced with a stressful factor such as the COVID-19 pandemic, based on experiences throughout their lives, potentially adapting in a healthier manner to adverse circumstances^(26,27)^.

In our study, bisexual orientation was a risk factor for both anxiety and non-psychotic disorders. Non-monosexual individuals (e.g., bisexual, pansexual) experience minority stressors associated with belonging to a minority within the sexual community (e.g., biphobia)^(24)^. Previous studies identified higher rates of mental disorders in bisexuals, possibly because of their invisibility in society^(28)^.

Furthermore, as expected, our findings indicated high rates of anxiety and minor mental (non-psychotic) disorders in the participants who reported previous medical conditions. Specifically, sexual and gender minority groups suffer from additive stress for having a marginalized identity. Moreover, they are subject to discrimination and prejudice from their families, educational institutions, religious communities, and broader society. Therefore, the COVID-19 pandemic intensifies ­pre-existing inequalities^(23,25)^.

We also found higher scores of anxiety and minor (­non-psychotic) mental disorders in participants with access to public health services only. Healthcare access problems were intensified during the COVID-19 pandemic and several public services were disrupted, affecting both access to diagnosis and treatment of well-known diseases and coping with COVID-19^(21,29)^. Moreover, sexual and gender minorities historically have difficulties accessing health services, or have suffered some kind of prejudice in these spaces, a factor that increases the fear of getting sick in Brazil^(29)^.

Another interesting finding of our study was the lower rate of minor (non-psychotic) mental disorders among people who held some religious beliefs than in those who profess no religion. In difficult situations such as a pandemic that directly affects the entire population, some resources like religious beliefs can be an emotional control tool^(20)^. Religion is a survival strategy for maintaining faith and hope. In the LGBTQIAP+ population, experiencing spirituality can contribute to facing physical and mental issues, directly influencing on their mental health^(26,30)^, especially when they are part of open and non-judgmental religious groups.

Some particularities related to mental health, such as stigma, prejudice, and discrimination, affect this population. Therefore, it is essential to know the situation and provide a safe and inclusive care environment, addressing issues such as lack of family support, difficulty in accessing health care, lack of understanding or acceptance by healthcare providers, and other barriers.

Our research had limitations that must be addressed. In the first place, all regions of the country were represented in the study, although unequally, with a large representation from the Brazilian South region. Online studies may limit access for people with low income and schooling levels due to the difficulty of internet access. This is a study with a cross-sectional design; thus, we are unaware of the prevalence of mental health problems in this sample before the pandemic. Finally, the utilization of an online questionnaire, especially across different platforms such as Instagram, WhatsApp, and Facebook, introduces a noteworthy selection bias - they may select similar participants, narrowing the variability.

## CONCLUSION

The results of the present study show a high prevalence of anxiety symptoms and minor non-psychotic disorders in the LGBTQIAP+ population, especially in younger individuals.

This study was innovative as a national survey to identify the frequency and factors associated with mental health in the LGBTQIAP+ community during the pandemic. The impacts were enhanced due to high vulnerability, the historical context, and the worsening health disparities. Considering the significant prevalence of anxiety symptoms and minor (non-psychotic) mental disorders, our findings show the necessity to raise awareness regarding mental health and develop health policies for these population segments.

Furthermore, nursing can also play an important role in promoting mental health through educational and preventive activities, such as raising awareness of mental health issues among the LGBTQIAP+ population and promoting acceptance and understanding of sexual and gender diversity.
